# Causal pathways of cerebral palsy in individuals with congenital anomalies: A cardiologist's perspective

**DOI:** 10.1111/dmcn.16077

**Published:** 2024-09-05

**Authors:** Mads Damkjær

**Affiliations:** ^1^ Department of Paediatrics and Adolescent Medicine Lillebaelt Hospital, University Hospital of Southern Denmark Kolding Denmark

## Abstract

This commentary is on the original article by Reid et al. on pages 374–381 of this issue.

Osler coined the term cerebral palsy (CP) in 1889^1^ and for 135 years it has been a recognized diagnostic entity. Hence, one could be forgiven for assuming it is a well‐defined entity. However, what lies beneath the seemingly well‐defined group of clinical disorders is a vastly more complex picture where a host of prenatal risk factors, intrauterine exposures, congenital anomalies, birth complications, and postnatal insults either alone or in unison may give rise to the clinical picture of CP. Gaining insights into the pathogenic pathways of CP is crucial for improving prevention strategies for at‐risk infants and managing affected individuals. However, this effort is complicated by the fact that the clinical presentation of CP encompasses a variety of distinct disease entities.

Reid et al. try to grapple with exactly this problem. In their elegant study, they investigated an Australian cohort of children with CP in whom they accessed electronic medical records and magnetic resonance imaging (MRI).^2^ To allow for comparison with recent studies from Europe, congenital anomalies were classified according to EUROCAT standards,^3^ a point which adds further strength and generalizability to their study. The MRI was then reviewed so that causal pathways of CP could be categorized into either: (1) developmental; (2) late prenatal/perinatal destructive insults; and (3) brain insults that occurred postneonatally, from the classification system proposed by Himmelmann et al.^4^

This approach offered new valuable insights into the causal pathways of CP in children with congenital anomalies. The authors identified that one‐quarter of children with CP had a congenital anomaly. Broadly, congenital anomalies in two organ systems appear to be on the causal pathway to CP – those are cerebral and cardiac anomalies. Interestingly, whereas the cerebral anomalies appear to directly influence CP development (through cortical malformation) the MRI findings in the patients with cardiac defects were suggestive of perinatal/postnatal insults to the central nervous system. Some of these could perhaps be a direct consequence of the cardiac defect (i.e. embolic stroke in a patient with right‐to‐shunt), but more likely these are related to the management of the cardiac defect. The past 50 years have seen great advances in the management of patients with congenital heart defects, so that overall survival for severe congenital heart defects is close to 90%, with fairly uniform timing and outcomes across Europe.^5^ As outcome goals for management of severe congenital heart defects moves towards a focus on long‐term morbidity, complications such as CP become increasingly important end‐points.

The paper by Reid et al. helps highlight that CP in individuals with severe congenital heart defects might be an avoidable complication through optimized management. Future studies on surgical interventions in children with severe congenital heart defects should, therefore, prioritize long‐term morbidities like CP as key endpoints in comparative trials of surgical strategies.

## Data Availability

Not required.
